# Progressive Deactivation of Hydroxylases Controls Hypoxia-Inducible Factor-1α-Coordinated Cellular Adaptation to Graded Hypoxia

**DOI:** 10.34133/research.0651

**Published:** 2025-04-01

**Authors:** Ping Wang, Xiao-Peng Zhang, Feng Liu, Wei Wang

**Affiliations:** ^1^ Kuang Yaming Honors School, Nanjing University, Nanjing 210023, China.; ^2^ Key Laboratory of High Performance Scientific Computation, School of Science, Xihua University, Chengdu 610039, China.; ^3^ Institute of Brain Sciences, Nanjing University, Nanjing 210093, China.; ^4^ National Laboratory of Solid State Microstructures and Department of Physics, Nanjing University, Nanjing 210093, China.

## Abstract

Graded hypoxia is a common microenvironment in malignant solid tumors. As a central regulator in the hypoxic response, hypoxia-inducible factor-1 (HIF-1) can induce multiple cellular processes including glycolysis, angiogenesis, and necroptosis. How cells exploit the HIF-1 pathway to coordinate different processes to survive hypoxia remains unclear. We developed an integrated model of the HIF-1α network to elucidate the mechanism of cellular adaptation to hypoxia. By numerical simulations and bifurcation analysis, we found that HIF-1α is progressively activated with worsening hypoxia due to the sequential deactivation of the hydroxylases prolyl hydroxylase domain enzymes and factor inhibiting HIF (FIH). Bistable switches control the activation and deactivation processes. As a result, glycolysis, immunosuppression, angiogenesis, and necroptosis are orderly elicited in aggravating hypoxia. To avoid the excessive accumulation of lactic acid during glycolysis, HIF-1α induces monocarboxylate transporter and carbonic anhydrase 9 sequentially to export intracellular hydrogen ions, facilitating tumor cell survival. HIF-1α-induced miR-182 facilitates vascular endothelial growth factor production to promote angiogenesis under moderate hypoxia. The imbalance between accumulation and removal of lactic acid in severe hypoxia may result in acidosis and induce cell necroptosis. In addition, the deactivation of FIH results in the destabilization of HIF-1α in anoxia. Collectively, HIF-1α orchestrates the adaptation of tumor cells to hypoxia by selectively inducing its targets according to the severity of hypoxia. Our work may provide clues for tumor therapy by targeting the HIF-1 pathway.

## Introduction

Hypoxia is a hallmark of the tumor microenvironment. In advanced solid tumors, malignant proliferation of tumor cells progressively distances themselves from blood vessels, thereby exacerbating hypoxia [1]. In the bone marrow, the oxygen partial pressure along individual blood vessels decreases gradually, indicating the presence of a hypoxic gradient [2]. Histological analysis of tissues from different cancer patients exhibits some typical properties including malignant proliferation, central necroptosis, and peripheral microvascular proliferation [3–5]. These heterogeneous morphological features may result from differential adaptive responses of tumor cells to graded hypoxia, as demonstrated in 3-dimensional tumor spheroid models [6]. However, the molecular mechanisms underlying tumor cell adaptation to varying levels of hypoxia remain less understood.

Hypoxia-inducible factor-1 (HIF-1), composed of oxygen-regulated HIF-1α and constitutively expressed HIF-1β, plays a key role in the cellular response to hypoxia [7,8]. In hypoxia, HIF-1α accumulates in cells and acquires transcriptional activity [9]. As a transcription factor, it can induce a series of target genes to mediate various cellular processes including glycolysis, angiogenesis, drug resistance, necroptosis, and cell migration [10]. It is a substantial challenge to understand how tumor cells exploit HIF-1α to orchestrate different responses to survive hypoxia.

The stability and transcriptional activity of HIF-1α are modulated by hydroxylases PHDs (prolyl hydroxylase domain enzymes) and FIH (factor inhibiting HIF). In normoxia, PHDs utilize oxygen and 2-oxoglutarate as co-substrates to hydroxylate HIF-1α on P402/P564 in the N-terminal transcriptional domain, leading to its degradation by von Hippel-Lindau (VHL) [11–13]. The transcriptional activity of HIF-1α is suppressed by FIH, which hydroxylates it on Asn803 in the C-terminal transcriptional domain (C-TAD) and prevents it from binding to transcriptional cofactors p300/CBP [14]. The independent regulation of stability and activity of HIF-1α by PHDs and FIH highlights their nonredundant roles in modulating HIF-1α function [15]. Under hypoxia, PHDs and FIH deactivate at different oxygen concentrations, leading to the progressive activation of HIF-1α [16]. It remains to be elucidated how PHDs and FIH coordinate to control HIF-1α activity and its selective induction of targets under hypoxia.

Increasing experimental evidence underscores the role of microRNAs (miRNAs) in regulating HIF-1α signaling [17,18]. In particular, miR-155, miR-210, and miR-182 are induced by HIF-1α and, in turn, regulate it via PHDs and FIH [19–22]. Among them, HIF-1α induces miR-182 to reduce the expression of PHD-2 and FIH, thereby amplifying its activation by positive feedback loops. On the other hand, HIF-1α up-regulates PHD-2 to repress its own activation, forming a negative feedback loop. Considering these feedback loops may add another layer of complexity to HIF-1α signaling. It is intriguing to clarify how feedback loops synergize to modulate the response of HIF-1α to hypoxia.

Tumor cells exploit HIF-1α signaling to adapt to hypoxia through multiple strategies. A remarkable adaptive response induced by HIF-1α is to increase glycolysis, compensating for reduced oxidative phosphorylation in energy metabolism [23]. HIF-1α promotes the shift to anaerobic metabolism by inducing various glycolytic enzymes, while decreasing mitochondrial oxygen consumption by up-regulating pyruvate dehydrogenase kinase 1 (PDK1) to attenuate the tricarboxylic acid (TCA) cycle [10,24–26]. However, this strategy alone would bring about a serious threat to cell survival. Lactic acid is a typical by-product of glycolysis. Excessive accumulation of intracellular acid leads to acidosis, triggering cell necroptosis in a BNIP3-dependent manner [27,28]. In addition, HIF-1α can alleviate hypoxic dilemmas by promoting immunosuppression [29] and angiogenesis [30]. It is still challenging to unravel the mechanism by which hypoxic cells choose among different outcomes based on the severity of hypoxia.

To relieve the survival stress caused by the accumulation of lactic acid, HIF-1 activates several proton exchangers and ion channels to produce an alkaline intracellular environment. Monocarboxylate transporter (MCT) and carbonic anhydrase 9 (CA9) are 2 representative targets of HIF-1, with the former responsible for exporting hydrogen ions and the latter contributing to intracellular H^+^ neutralization [27]. Notably, this acidic extracellular environment confers a growth advantage to cancer by inducing cell growth [31], blunting the immune system [32] and promoting epithelial–mesenchymal transition (EMT) involved in metastasis [33]. That is, HIF-1 acts as a potent regulator of the redistribution of intracellular and extracellular acids. It remains unclear how HIF-1 balances adenosine triphosphate (ATP) reduction and excessive lactic acid during the formation of an acidic microenvironment.

A series of models has been developed to reveal the mechanism underlying the modulation of HIF-1α dynamics. As the oxygen level decreases, HIF-1α appears to have a switch-like behavior; i.e., it remains low at wide oxygen levels and increases exponentially to high concentrations at narrow oxygen levels [34]. Qutub et al. [35] demonstrated that saturated substrates required for PHD-2 catalysis contribute to switch-like reactions, and the presence of any molecular unsaturation in these substrates results in a progressive response of HIF-1α to hypoxia. Bagnall et al. [36] proposed that the adaptive dynamics of HIF-1α in response to hypoxia is controlled by the HIF-1α-PHD negative feedback loop. Nguyen et al. [37] found that the remarkable decrease in HIF-1α levels in severe hypoxia should be associated with the destabilization of HIF-1α without the protection of FIH-mediated hydroxylation. Although most models have focused on the mechanism underlying the generation of HIF-1α dynamics, a comprehensive model of how different cellular processes are regulated by the HIF-1α dynamics is still lacking.

Here, we developed a network model to explore how HIF-1α mediates the adaptive response of tumor cells to hypoxia. We probed how cellular outcome was associated with network dynamics, the regulation of HIF-1α activity by PHD-2 and FIH, the role of miR-182 in modulating HIF activity and angiogenesis, and how the accumulation of lactic acid and its intracellular/extracellular distribution were modulated. We found that the coupled feedback loops involving HIF-1α, miR-182, FIH, and PHD-2 determine the selective induction of HIF-1 targets, linking the circuit regulation of HIF-1α with its physiological functions.

## Results

### Network model

**Fig. 1.**
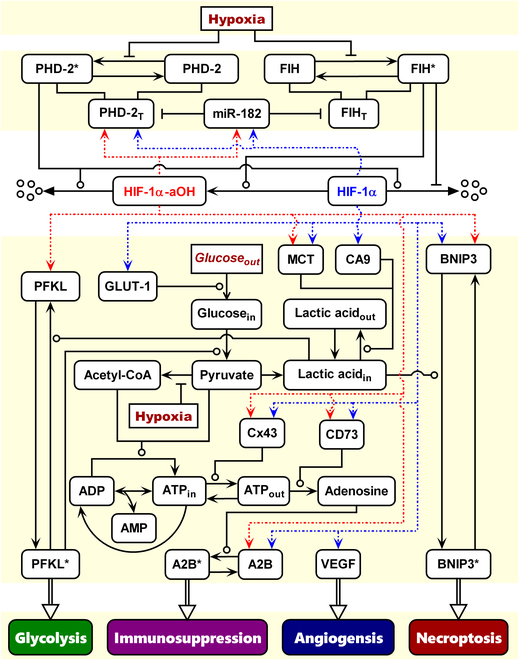
Schematic of the integrated model. Upstream, the coupled HIF-1α-PHD-2 and HIF-1α-miR-182-PHD-2/FIH feedback loops regulate the stability and activity of HIF-1α. Downstream, PFKL, A2B, VEGF, and BNIP3 are indicators of altered energy metabolism, immunosuppression, angiogenesis, and necroptosis, respectively. HIF-1α-mediated transactivation of target genes is denoted by dashed lines. State transitions are labeled by arrow-headed solid lines. The promotion and repression of state transitions or interactions between proteins are represented by round- and bar-headed lines, respectively. Other processes are marked by hollow arrows.

We developed an integrated model of the HIF-1α network and explored the network dynamics and cellular adaptive response under hypoxia (Fig. 1). PHD-2, FIH, and miR-182 are involved in the modulation of the stability and activity of HIF-1α under hypoxia. phosphofructokinase liver type (PFKL), A2B, vascular endothelial growth factor (VEGF), and BNIP3 are 4 effectors of HIF-1α, whose high expressions and/or activation represent the initiation of glycolysis, immunosuppression, angiogenesis, and necroptosis, respectively. The details of model construction are presented as follows.

#### Regulation of HIF-1α by PHD-2 and FIH

The prolyl hydroxylase family mainly consists of PHD-1, PHD-2, and PHD-3. PHD-1 and PHD-2 are localized in the nucleus and cytoplasm, respectively, while PHD-3 is present in both compartments [38]. Since PHD-1 and PHD-3 preferentially target HIF-2α [39] and PHD-2 has a remarkable advantage over PHD-1/PHD-3 in hydroxylating HIF-1α [40], we considered the regulation of HIF-1α by PHD-2 alone. FIH promotes the hydroxylation of HIF-1α on Asn803, inhibiting its transcriptional activity while repressing its PHD-independent degradation [37].

Two forms of HIF-1α were considered according to their hydroxylation status associated with their activity. Considering that C-TAD-truncated HIF-1α retains one-third of its full transcriptional activity [41], it was assumed that HIF-1α-aOH is the partially activated form that is hydroxylated on Asn803 in C-TAD alone. The entirely dehydroxylated HIF-1α was considered the fully activated form. Moreover, PHD-2 and FIH were divided into inactive (PHD-2 and FIH) and active (PHD-2^*^ and FIH^*^) forms. The activation of the 2 hydroxylases depends on oxygen levels. Based on experimental results, we assumed that the threshold of oxygen levels for FIH activation ($j_{O2\mathrm{FIH}}$) is much lower than that for PHD-2 ($j_{O2\mathrm{PHD}}$) since PHD-2 is deactivated before FIH with worsening hypoxia [16] (see Eqs. 5 and 8 in the Supplementary Materials). The total amounts of PHD-2 and FIH are represented by PHD-2_Τ_ and FIH_Τ_, respectively.

Both HIF-1α-aOH and HIF-1α were assumed to be degraded in PHD-2^*^-dependent and -independent ways (see Eqs. 1 and 2 in the Supplementary Materials). For simplicity, the role of pVHL is implicated in PHD-2-dependent degradation of HIF-1α in which pVHL targets the hydroxylated form of HIF-1 for ubiquitination [11–13]. FIH^*^ promotes the transition from HIF-1α to HIF-1α-aOH while inhibiting their PHD-2^*^-independent degradation [37]. PHD-2 is induced by both HIF-1α and HIF-1α-aOH, characterized by Hill functions (see Eq. 4 in the Supplementary Materials). The oxygen-dependent activation of hydroxylases (PHD-2 and FIH) and FIH^*^-dependent inhibition of HIF-1α degradation obey the Michaelis-Menten kinetics (see Eqs. 1, 5, and 8 in the Supplementary Materials).

#### Repression of PHD-2 and FIH production by miR-182

Although the role of miR-182 in tumor development is controversial, its carcinogenic function by promoting HIF-1α activation has been widely reported [20,21,42]. Typically, miR-182 targets the 3′-untranslated region of PHD-2 and FIH to activate HIF-1 indirectly [21], while HIF-1α induces miR-182 production, creating 2 positive feedback loops involving HIF-1α, PHD-2, FIH, and miR-182. Regardless of their status, PHD-2 and FIH were assumed to be down-regulated by miR-182 (see Eqs. 4 and 7 in the Supplementary Materials). Since hypoxia-dependent miR-182 up-regulation was found at 1% O_2_ within the range of oxygen levels for HIF-1α-aOH activation [21], we assumed that both HIF-1α-aOH and HIF-1α can promote miR-182 production (see Eq. 3 in the Supplementary Materials).

#### Regulation of cellular pH by HIF-1α

The pH level of internal and external environments of tumor cells is regulated by HIF-1α in diverse manners. HIF-1α can lower pH by accelerating glycolysis, which leads to considerable accumulation of intracellular lactic acid. The production of glycolytic enzymes like PFKL and glucose transporters like GLUT1 is mediated by HIF-1α [10,43]. HIF-1α also scavenges intracellular H^+^ by inducing MCT and CA9 since the former promotes the export of lactic acid and the latter neutralizes intracellular H^+^ via increasing the production of HCO_3_^−^ [44,45].

We simplified the characterization of lactic acid production, focusing on the underlying regulatory mechanism. Cellular uptake of glucose is facilitated by GLUT1, and active PFKL boosts the production of lactic acid from glucose. Lactic acid_in_ (abbreviation, LA_in_) and Lactic acid_out_ (abbreviation, LA_out_) represent the intracellular and extracellular lactic acid, respectively. MCT and CA9 promote the accumulation of LA_out_. Since GLUT1 and CA9 are the FIH-inhibited targets of HIF-1α [27], their production rates were assumed to be proportional to the Hill function of the HIF-1α level (see Eqs. 15 and 11 in the Supplementary Materials). Because MCT was found to be induced at 1% O_2_ [46], it was assumed to be induced by both HIF-1α-aOH and HIF-1α (see Eq. 12 in the Supplementary Materials).

#### Determination of different cellular outcomes

HIF-1α can induce glycolysis, immunosuppression, tumor angiogenesis, and necroptosis in response to hypoxia. As the critical rate-limiting enzyme involved in the only irreversible step in glycolysis, PFKL controls the conversion from fructose-6-P phosphorylation to fructose-1,6-bisphosphate [47]. Its enzymatic activity is highly dependent on pH and can be reduced by lowering the pH through allosteric regulation [48]. A slightly alkaline intracellular environment is optimal for maximizing PFKL activity [49]. Here, we differentiated 2 forms of PFKL, PFKL (inactive) and PFKL^*^ (active), and their conversion is regulated by LA_in_ (Eqs. 8 and 9 in the Supplementary Materials).

Hypoxia also facilitates tumor cells to acquire immunosuppressive traits by pumping intracellular ATP out of the cells [50–52]. In the extracellular space, ATP is converted into adenosine monophosphate (AMP) by ectonucleoside triphosphate diphosphohydrolase 1 (CD39) and further catalyzed by ecto-5′-nucleotidase (CD73) to generate adenosine [53]. Studies show that excess extracellular adenosine activates the A2B adenosine receptor, promoting immunosuppression [54]. Furthermore, hypoxia enhances the expression of connexin 43 (Cx43) in tumor cell-derived exosomes, promoting ATP release [55–58]. Notably, Cx43 [57], CD73 [59], and A2B [60] are all target genes of HIF-1. We hypothesize that glucose is converted into pyruvate and acetyl-CoA to promote ATP production, while hypoxia inhibits the conversion of pyruvate into acetyl-CoA (Eqs. 18 to 20 in the Supplementary Materials). It was assumed that the interconversions among ATP, AMP, and ADP (adenosine diphosphate) follow the mass action law, and the total of their concentration is a constant ([AXP_t_]) (Eqs. 24 to 26 in the Supplementary Materials). In this context, ATP is transported to the extracellular space via Cx43, converted into adenosine by CD73, and ultimately activates the A2B receptor (Eqs. 27, 29, and 31 in the Supplementary Materials). Given that mild hypoxia could induce the expression of Cx43, CD73, and A2B [57,59,60], the expression of these genes was assumed to be regulated by both HIF-1α and HIF-1α-aOH (Eqs. 23, 28, and 30 in the Supplementary Materials). Additionally, we assumed that adenosine promotes the conversion of A2B from its inactive to active form (Eqs. 30 and 31 in the Supplementary Materials).

HIF-1α induces VEGF and angiopoietin-2 (Ang-2) to regulate angiogenesis by directing the migration of mature endothelial cells to the hypoxic zone [61]. At the tip of the sprouts, endothelial cells use long filopodia in the receptors to sense VEGF [62]. Ang-2 is responsible for blocking Tie-2 signaling and increasing endothelial permeability to allow VEGF-induced cell migration [63]. For simplicity, we only considered VEGF as an effector in HIF-1α-dependent angiogenesis. Since VEGF belongs to the FIH-inhibited targets of HIF-1α [27], its synthesis is induced by fully activated HIF-1α alone (Eqs. 10 and 12 in the Supplementary Materials).

The hypoxic tumor tissue far from blood vessels shows a necrotic morphology [3]. This is mediated by HIF-1α-induced BNIP3, a member of non-FIH-inhibited targets [15]. Although mild hypoxia is sufficient to induce BNIP3 overexpression, cells are necrotic only under severe hypoxia. This mechanism may be involved in intracellular acidosis, which not only improves the stability of BNIP3 but also enhances its binding to the mitochondrial membrane, thereby activating its pronecrotic activity [28]. It was assumed that both forms of HIF-1α were responsible for BNIP3 induction. We considered 2 forms of BNIP3, i.e., BNIP3 (inactive form) and BNIP3^*^ (active form), and the conversion from BNIP3 to BNIP3^*^ is promoted by LA_in_ (Eqs. 13 and 14 in the Supplementary Materials).

### Diverse cellular outcomes mediated by HIF-1α

**Fig. 2.**
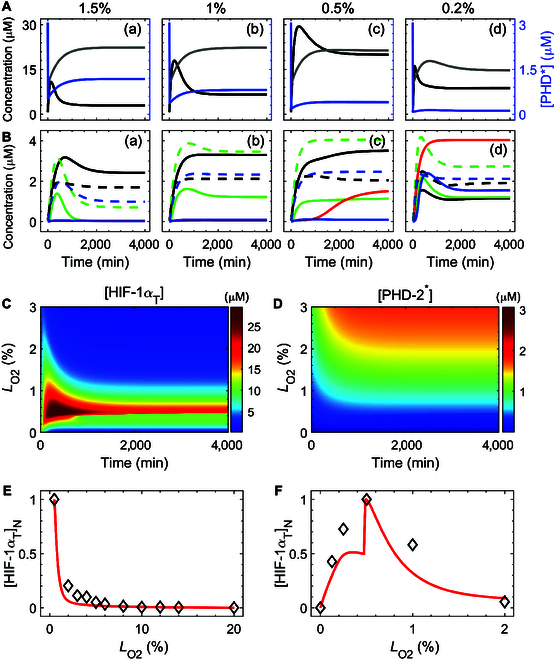
Hypoxia-dependent cellular response mediated by HIF-1α. (A and B) Time courses of [HIF-1α_Τ_] (black), [PHD-2^*^] (blue), and [PHD-2_T_] (gray) (A), and of [PFKL] (black and dashed), [PFKL^*^] (black and solid), [A2B] (green and dashed), [A2B^*^] (green and solid), [VEGF] (red), [BNIP3] (blue and dashed), and [BNIP3^*^] (blue and solid) (B) for *L*_O2_ = 1.5% (a), 1% (b), 0.5% (c), and 0.2% (d). (C and D) Color-coded concentrations of HIF-1α_Τ_ (C) and PHD-2^*^ (D) as a function of *L*_O2_ and time. (E and F) Steady-state value of [HIF-1α_Τ_] normalized by its maximum versus *L*_O2_ [0.5% to 20% (E) and 0% to 2% $O_{2}$ (F)]. Red lines denote simulation results, while black squares refer to the experimental data from HeLa cells at 4 h [34]. [HIF-1α_T_] indicates the total of [HIF-1α-aOH] and [HIF-1α]. The symbol [...]_N_ stands for normalized concentration.

To explore the adaptability of tumor cells to graded hypoxia, we showed the dynamics of different signaling components in the cellular response to hypoxia (*L*_O2_ ≤ 3%). Under mild hypoxia (e.g., *L*_O2_ = 1.5%), HIF-1α accumulated and exhibited bell-shaped dynamics [Fig. 2A(a)]. Its total concentration, [HIF-1α_T_], rapidly rose to a peak and then slowly returned to a level marginally higher than its initial expression. By contrast, [PHD-2^*^] dropped dramatically and then rose gradually toward saturation at a lower level. The restoration of [PHD-2^*^] was caused by the transcriptional up-regulation of PHD-2 by HIF-1α. In this case, the levels of active PFKL^*^ and inactive A2B and BNIP3 rose prominently, while active A2B^*^ increased initially but returned to basal levels over time, and VEGF and active BNIP3^*^ remained at basal levels [Fig. 2B(a)]. This corresponds to a change in cellular metabolism from aerobic to anaerobic glycolysis. Indeed, compensating for oxygen deficiency by accelerating ATP production is a canonical adaptive response and is frequently preyed upon by tumors [64].

When the oxygen level decreased from 1.5% to 1%, PHD-2 activity further diminished, leading to a further increase in HIF-1α levels while maintaining the bell-shaped curve [Fig. 2A(b)]. While the moderate elevation in HIF-1α did not notably change the low expression levels of VEGF and BNIP3^*^, it elevated the levels of PFKL and A2B^*^ markedly, indicating that cells could start to initiate immunosuppressive functions [Fig. 2B(c)].

Under moderate hypoxia (e.g., *L*_O2_ = 0.5%), [HIF-1α_T_] was elevated remarkably compared with the case of mild hypoxia, while [PHD-2^*^] was down-regulated despite a slight change in [PHD-2_Τ_] [Fig. 2A(c)]. Thus, [PFKL^*^] increased further and [VEGF] rose moderately [Fig. 2B(c)]. Consequently, angiogenesis was triggered to promote tumor survival. This result suggests that in response to the aggravated loss of oxygen, cells would resort to additional strategies to adapt to hypoxia.

Under severe hypoxia (e.g., *L*_O2_ = 0.2%), all 3 curves shifted downward compared with the case of *L*_O2_ = 0.5% [Fig. 2A(d)]. The down-regulation of HIF-1 may be associated with its destabilization due to the deactivation of FIH [37]. However, [A2B], [VEGF], and [BNIP3^*^] were still maintained at relatively high levels in contrast to low levels of [PFKL^*^] after the transients [Fig. 2B(d)]. Notably, BNIP3 was always strongly induced under hypoxia (Fig. 2B, blue) but was highly activated only during severe hypoxia, leading to cell necroptosis [Fig. 2B(d)] [45]. The coexistence of highly expressed VEGF and BNIP3^*^ is consistent with the report that necroptosis provides a spatial structure for early angiogenesis [4].

Moreover, the color-coded time courses of [HIF-1α_T_] and [PHD-2^*^] across *L*_O2_ were exploited to show the dependency of their dynamics on the extent of hypoxia (Fig. 2C and D). For *L*_O2_ > 1%, [HIF-1α_T_] showed adaptive dynamics, while [PHD-2^*^] remained at high levels; for $0.5\%\leq L_{O2}\leq 1\%$, [HIF-1α_T_] reached rather high levels since PHD-2^*^ dropped to low levels. In addition, we showed the dependency of the steady-state level of HIF-1α_T_ on *L*_O2_ (Fig. 2E and F). [HIF-1α_T_] remained low for *L*_O2_ > 2%, ensuring its inactivation under abundant oxygen; for $0.5\%\leq L_{O2}\leq 2\%$, [HIF-1α_T_] rose exponentially with decreasing *L*_O2_, indicating its sensitivity to oxygen deficiency (Fig. 2E). When *L*_O2_ decreased from 2% to 0%, [HIF-1α_T_] rose to its peak around 0.5% $O_{2}$ followed by dropping to its basal levels due to its degradation in severe hypoxia (Fig. 2F) [37]. Our results are in good concordance with the experimental data [34].

Taken together, our model allows for the fine-tuning of HIF-1α dynamics, leading to an ordered selection of cellular outcomes depending on the severity of hypoxia. This selectivity may account for the morphological distribution of hypoxic tissues at different distances from blood vessels [3]. A broad area of tumor tissue is located close to the blood vessels, while the tumor tissue distant from the blood tissues undergoes necroptosis. Consistently, VEGF is mainly expressed around necrotic areas [65].

### The hydroxylation status of HIF-1α determines its selection for different targets

**Fig. 3.**
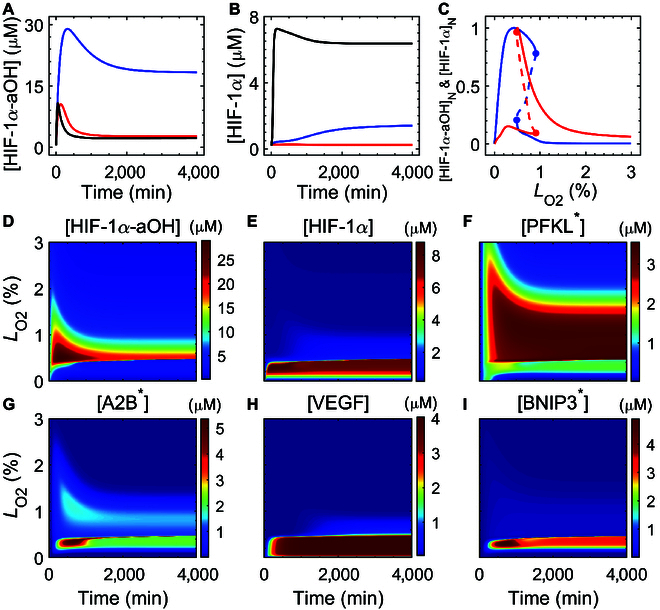
The hydroxylation status of HIF-1α determines the selection of cellular outcome. (A and B) Temporal trajectories of [HIF-1α-aOH] (A) and [HIF-1α] (B) for *L*_O2_ = 1.5% (red), 0.5% (blue), and 0.2% (black). (C) Bifurcation diagrams of normalized [HIF-1α] (blue) and [HIF-1α-aOH] (red) versus *L*_O2_. The stable and unstable steady states are denoted by solid and dashed lines, respectively. The subscript “N” indicates normalization. (D to I) Color-coded [HIF-1 α-aOH] (D), [HIF-1α] (E), [PFKL^*^] (F), [A2B^*^] (G), [VEGF] (H), and [BNIP3^*^] (I) as a function of *L*_O2_ and time.

HIF-1α-aOH and HIF-1α are partially and fully activated forms of HIF-1α, respectively, with different transcriptional activity. Their expression levels differed remarkably and predominated in different regimes of *L*_O2_. [HIF-1α-aOH] was higher under mild and moderate hypoxia but declined to low levels under severe hypoxia (Fig. 3A). [HIF-1α-aOH] exhibited an adaptive response in each case, resulting from the negative feedback between HIF-1α-aOH and PHD-2. By contrast, [HIF-1α] was kept at low levels under mild and moderate hypoxia; it rose to intermediate levels under severe hypoxia (Fig. 3B).

To systematically show the dependence of HIF-1α expression on oxygen deficiency, we plotted the bifurcation diagrams of normalized [HIF-1α-aOH] and [HIF-1α] versus *L*_O2_ (Fig. 3C). There existed 2 saddle-node (SN) bifurcation points, implying that the dynamics of HIF-1α were governed by a bistable switch when *L*_O2_ was varied. With decreasing *L*_O2_, [HIF-1α-aOH] rose along the upper branch; at SN_1_, it jumped down to the lower branch. By contrast, [HIF-1α] climbed first along the lower branch until SN_1_ and then switched to the upper branch. Of note, [HIF-1α] dropped to very low levels from the peak of the upper branch under anoxia due to the destabilization of HIF-1α. Thus, *L*_O2_ at SN_1_ was a threshold for differentiating the predominance of HIF-1α-aOH over HIF-1α. These results further confirm that HIF-1α was activated progressively: HIF-1α-aOH was gradually activated under mild and moderate hypoxia, while HIF-1α was induced in severe hypoxia.

We further displayed the color-coded time courses of key molecules across *L*_O2_ in Fig. 3D to I. For $0.5\%\leq L_{O2}\leq 2\%$, HIF-1α-aOH was of sufficient expression, accompanied by a significant increase in both of [PFKL^*^] and [A2B^*^]. The response range of [PFKL^*^] was broader than that of [A2B^*^], suggesting that the cell first adjusts its energy metabolism and then initiates immunosuppression functions. [HIF-1α], [VEGF], and [BNIP3^*^] all remained at relatively high values for $0.2\%\leq L_{O2}<0.5\%$, corresponding to the induction of angiogenesis and necroptosis. [HIF-1α] was kept at rather low levels for *L*_O2_ < 0.2%, and apoptosis may be induced by the tumor suppressor p53, as reported in Ref. [66]. Thus, anaerobic glycolysis is first adopted by tumor cells to survive mild hypoxia, then angiogenesis is initiated to withstand moderate hypoxia, and necroptosis accompanied by angiogenesis is induced under severe hypoxia.

### PHD-2 and FIH differentially regulate the hydroxylation status of HIF-1α

**Fig. 4.**
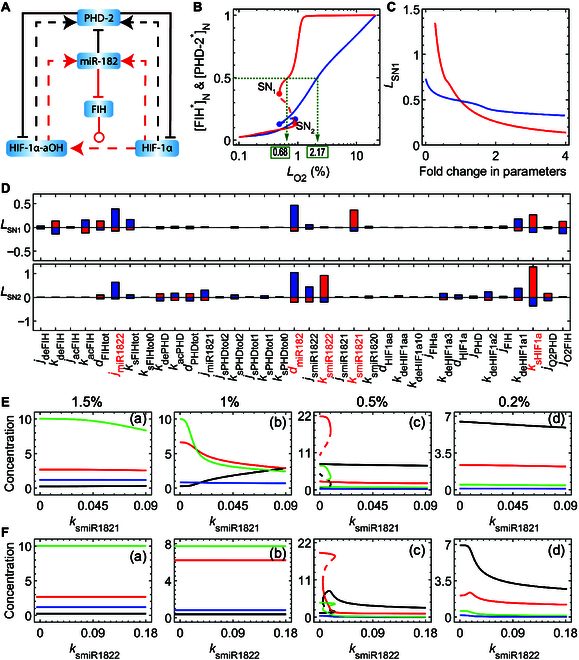
Sequential induction of HIF-1α-aOH and HIF-1α by PHD-2 and FIH. (A) Simplified schematic of the interconnected HIF-1α/FIH/PHD-2/miR-182 loops. (B) Bifurcation diagrams for [FIH^*^] (red) and [PHD-2^*^] (blue) versus *L*_O2_. [FIH^*^] and [PHD-2^*^] decay to half their initial concentrations at 0.68% and 2.17% O_2_, respectively. (C) *L*_SN1_ as a function of fold change in *k*_acPHD_ (blue) or *k*_acFIH_ (red). (D) Percentage changes of *L*_SN1_ and *L*_SN2_ as each of the 36 parameters characterizing the coupled HIF-1α/FIH/PHD-2/miR-182 loops increases or decreases by 15% relative to the default value. (E and F) Bifurcation diagram of HIF-1α-aOH (red), HIF-1α (black), PHD-2^*^ (blue), and FIH^*^ (green) versus *k*_smiR1821_ (E) and *k*_smiR1822_ (F) for *L*_O2_ = 1.5% (a), 1% (b), 0.5% (c), and 0.2% (d).

PHD-2 and FIH determine the hydroxylation status and activity of HIF-1α; it was worthwhile to clarify the underlying mechanism. Analysis of the network model revealed that the activity and concentration of these 2 hydroxylases were regulated by oxygen levels and their interaction network with miR-182 (Fig. 4A). In the bifurcation diagrams for [FIH^*^] and [PHD-2^*^] (Fig. 4B), there existed 2 SN bifurcation points. With decreasing *L*_O2_, [PHD-2^*^] declined almost linearly (on the logarithm scale) along the upper branch, while [FIH^*^] remained unchanged until 1.5% $O_{2}$ and then dropped quickly; they both varied gradually along the lower branches. Thus, PHD-2 was more sensitive to mild hypoxia than FIH. Indeed, [PHD-2^*^] reached half its maximum at 2.17% $O_{2}$, around which initial accumulation of HIF-1α was observed experimentally [34], whereas [FIH^*^] halved at 0.68% $O_{2}$, which was close to *L*_O2_ at SN_1_. Thus, the sequential inactivation of PHD-2 and FIH leads to the progressive activation of HIF-1α, and the transition involves the switch-like mechanism.

The oxygen concentration at SN_1_ (SN_2_) is denoted by *L*_SN1_ (*L*_SN2_). *L*_SN1_ corresponds to the threshold of *L*_O2_ for full activation of HIF-1α, which is influenced by the rate constants for PHD-2 and FIH activation, *k*_acPHD_ and *k*_acFIH_ (Fig. 4C). *L*_SN1_ rose slowly with increasing *k*_acPHD_, whereas it changed sharply with *k*_acFIH_. Thus, FIH (rather than PHD-2) could effectively modulate the transition from HIF-1α-aOH to HIF-1α. It was worth extending the above analysis to examine the sensitivity of *L*_SN1_ and *L*_SN2_ for FIH to changes in 36 parameter values (Fig. 4D). They were more sensitive to the following parameters: the rate constant of HIF-1 expression (*k*_sHIF1a_), rate constants involved in miR-182 regulation (*k*_smiR1821_, *k*_smiR1822_ and *d*_smiR182_), and the Michaelis constant of miR-182 for FIH inhibition (*j*_miR1822_); these parameters were all associated with the interconnected loops between HIF-1α, HIF-1α-aOH, miR182, and FIH (red lines in Fig. 4A). Of note, HIF-1α-aOH and HIF-1α induced miR-182 to repress FIH expression, creating interlinked negative and positive feedback loops; they promoted the expression of PHD-2, facilitating their own degradation and enclosing 2 negative feedback loops (Fig. 4A).

We further investigated the influence of 2 production rates of miR-182 (HIF-1α-aOH-dependent and HIF-1α-dependent production rates separately denoted by *k*_smiR1821_ and *k*_smiR1822_) on [HIF-1α-aOH] and [HIF-1α] with worsening hypoxia. An elevation in either *k*_smiR1821_ or *k*_smiR1822_ led to a marked reduction in [FIH^*^] and promoted the conversion of HIF-1α-aOH to HIF-1α [Fig. 4E(b and c) and F(c)]. Under severe hypoxia, an increase in *k*_smiR1822_ diminishes both [HIF-1α-aOH] and [HIF-1α] [Fig. 4F(d)]. This effect was due to a notable decrease in [FIH^*^] triggered by elevated [miR-182], which not only reduced the conversion rate of [HIF-1α] to [HIF-1α-aOH] but also compromised the stability of HIF-1α [Fig. 4F(d)]. Of note, the effects of the 2 production rates of miR-182 are negligible under different hypoxic conditions. Taken together, when other parameters take standard values, miR-182 mainly regulates HIF-1α via FIH instead of PHD-2.

### miR-182 acts as a sliding regulator to release FIH-inhibited HIF-1 target genes under moderate hypoxia

**Fig. 5.**
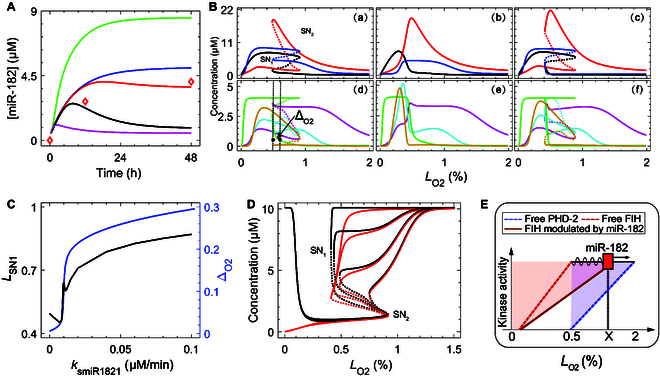
Fine-tuning of HIF-1α activity by miR-182. (A) Time courses of [miR-182] under 1.5% (black), 1% (red), 0.5% (blue), 0.2% (green), and 0% (magenta) $O_{2}$. Red squares represent the experimental data from DFO-treated PC-3 cells [21]. (B) Steady-state levels of [HIF-1α-aOH] (red, top), [HIF-1α] (black, top), and [miR-182] (blue, top), and [PFKL^*^] (magenta, bottom), [A2B^*^] (cyan, bottom), [VEGF] (green, bottom), and [BNIP^*^] (yellow, bottom) under default setting [(a) and (d)], *k*_smiR1822_ = 0 [(b) and (e)], and *k*_smiR1821_ = 0 [(c) and (f)]. (C) *L*_SN1_ (red) and Δ_O2_ (black) for [VEGF] as a function of *k*_smiR1821_. (D) Steady-state levels of [FIH^*^] (red) and [FIH_tot_] (black) for *k*_smiR1821_ = 0, 0.006, 0.009, and 0.012 (from left to right). (E) Schematic diagram depicting the role of miR-182 as a sliding regulator.

The expression of miR-182 heavily relied on HIF-1α dynamics under different hypoxic conditions (Fig. 5A). [miR-182] exhibited adaptive dynamics due to the adaptation of HIF-1α to mild hypoxia. Under moderate hypoxia (e.g., 1% O_2_), [miR-182] rose toward saturation, consistent with experimental data [21]. [miR-182] reached rather high levels under severe hypoxia (e.g., 0.2% O_2_) due to the full activation of HIF-1α. In contrast, it dropped to rather low levels in anoxia due to down-regulation of HIF-1α (see Fig. 3).

In the bifurcation diagram for [miR-182], 2 SN bifurcation points appeared at the same *L*_O2_ as for [HIF-1α] [Fig. 5B(a)]. Correspondingly, PFKL, A2B, and BNIP3 were activated sequentially due to the progressive activation of HIF-1α, while the expression of VEGE was initiated in an exclusive region of $0.5\%\leq L_{O2}\leq 1\%$ [Fig. 5B(d)]. The bifurcation diagrams were shaped by 2 transcription rates, *k*_smiR1821_ (by HIF-1α-aOH) and *k*_smiR1822_ (by HIF-1α), which separately modulated the left and right arms in the HIF-1α/HIF-1α-aOH-miR182-FIH feedback loops (Fig. 4D). When *k*_smiR1822_ was set to 0, i.e., blocking the HIF-1α-miR182-FIH positive feedback loop, the bistability was replaced by monostability, and [miR-182] took low levels for *L*_O2_ < 0.3% [Fig. 5B(b)]. As a result, [BNIP3^*^] exhibited monostability and was unable to remain high over a wide range of oxygen levels [Fig. 5B(e)], inconsistent with the scenario for terminating unrescuable cells (e.g., apoptosis) [67]. That is, sufficient induction of miR-182 by fully activated HIF-1α is critical for the bistability in miR-182 expression as well as necroptosis under severe hypoxia. By contrast, the steady-state level of A2B^*^ increased sharply till approximately 0.3 % $O_{2}$ [Fig. 5B(e)], indicating that under severe hypoxia, miR-182 loss may enhance HIF-1α expression by stabilizing FIH, which, in turn, drives a shift in cell fate from programmed necrosis to immunosuppression. This prediction could be experimentally validated by knocking out miR-182.

When *k*_smiR1821_ was set to 0, i.e., the left arm of the HIF-1α-aOH-miR182-FIH negative feedback loop was disrupted, the bistability remained intact in the bifurcation diagram, but the lower branch of [miR-182] fell markedly [Fig. 5B(c)], while VEGF was induced synergetically with BNIP3^*^ for *L*_O2_ < 0.5% [Fig. 5B(f)] and was down-regulated remarkably for $0.5\%\leq L_{O2}\leq 1\%$ compared with the normal case [Fig. 5B(d)]. That is, there was no exclusive *L*_O2_ range for HIF-1 to induce VEGF alone, which may decrease the possibility of tumor angiogenesis. The distance between *L*_SN1_ and *L*_O2_ at which [VEGF] reached one-fifth of its maximum was defined as $\Delta_{O2}$, characterizing the difference in *L*_O2_ between VEGF production and BNIP3 activation. $\Delta_{O2}$ rose with increasing *k*_smiR1821_ beyond 0.02, indicating that this exclusive induction of VEGF is greatly facilitated by the HIF-1α-aOH-mediated expression of miR-182 (Fig. 5C).

Notably, the divergence in VEGF expression and BNIP3 activation across *L*_O2_ ranges was related to the regulation of FIH by miR-182. Similar to [miR-182], both [FIH^*^] and [FIH_Τ_] showed bistability under moderate hypoxia (Fig. 5D). For *k*_smiR1821_ = 0, both [FIH_Τ_] and [FIH^*^] remained high along their upper branches and started to differ at 0.7% $O_{2}$; with increasing *k*_smiR1821_, the bifurcation point SN_1_ shifted rightward and the bistability range shortened, while the upper branches were closer to each other and shifted downward (Fig. 5D). As *L*_O2_ decreased, FIH underwent 2 layers of regulation: a miR-182-mediated decrease in concentration (0.7% < $L_{O2}<1.2\%$) followed by a hypoxia-mediated decrease in activity (<0.7%). Thus, progressive deactivation of FIH^*^ under worsening hypoxia leads to the gradual accumulation of fully activated HIF-1α, which sequentially induces VEGF and activates BNIP3.

Taken together, miR-182 can be considered a sliding regulator of the hypoxia switch. As shown in the cartoon (Fig. 5E), active FIH and PHD-2 were remarkably down-regulated around 0.5% and 2% $O_{2}$ (Fig. 4A), respectively, while miR-182 had a regulatory role over $0.5\%\leq L_{O2}\leq 1.2\%$ (Fig. 5D), promoting the functioning of fully activated HIF-1α, such as driving angiogenesis by destroying FIH.

### Hypoxia degree-dependent acidic microenvironment formation and acidosis-activated necroptosis

Tumor cells can create a survival-friendly acidic microenvironment under hypoxia, depending on the amount and distribution of lactic acid [45]. Excessive intracellular lactic acid can be lethal to hypoxic cells and lead them to necroptosis. [LA_in_] and [LA_out_] denote the concentrations of lactic acid inside and outside the cell, respectively. We hypothesized that sufficient LA_in_ could cause acidosis and thus necrotize cells, while sufficient LA_out_ implied the establishment of an acidic microenvironment.

**Fig. 6.**
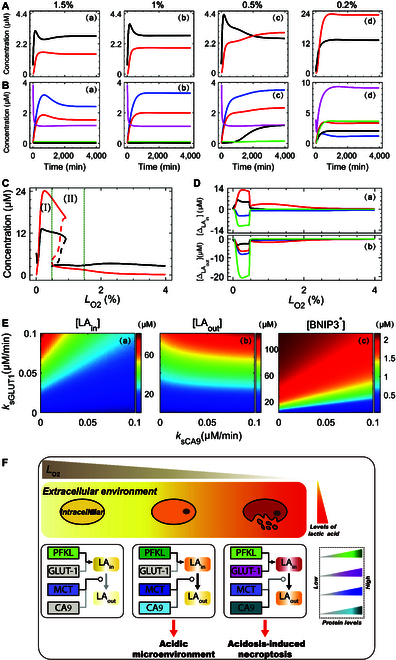
The level of lactic acid determines the formation of an acidic microenvironment or acidosis-induced necroptosis. (A and B) Time courses of [LA_in_] (black) and [LA_out_] (red) in (A), and [PFKL^*^] (blue), [CA9] (black), [MCT] (red), [GLUT-1] (green), and [Glucose] (magenta) in (B) for *L*_O2_ = 1.5% (a), 1% (b), 0.5% (c), and 0.2% (d). (C) Steady-state values of [LA_in_] (black) and [LA_out_] (red) versus *L*_O2_. (D) Effects of separately deleting PFKL (blue), CA9 (black), MCT (red), and GLUT1 (green) on steady-state values of [LA_in_] (a) and [LA_out_] (b). (E) Steady-state levels of LA_in_ (a), LA_out_ (b), and BNIP3^*^ (c) versus the parameter pair (*k*_sGLUT1_ and *k*_sCA9_) for *L*_O2_ = 0.2%. (F) Schematic of the cellular distribution of lactic acid under mild (1% < *L*_O2_ ≤ 2%), moderate (0.5% < *L*_O2_ ≤ 1%) and severe (*L*_O2_ < 0.5%) hypoxia (from left to right) and the corresponding activated pathways.

We first examined how the concentration and distribution of lactic acid were correlated with the severity of hypoxia. Under mild hypoxia, [LA_in_] was greater than [LA_out_] [Fig. 6A(a)] due to initially accelerated glycolysis characterized by a remarkable increase in [PFKL^*^] [Fig. 6B(a)]; the increase in [MCT] promoted the export of lactic acid. Under moderate hypoxia, [LA_out_] predominated in the later phase of the response [Fig. 6A(b)]. This was associated with increases in [PFKL^*^], [CA9], and [MCT]; the former induced the production of lactic acid, while the latter two promoted its extracellular localization to form an acidic microenvironment [Fig. 6B(b)].

Under severe hypoxia, both [LA_in_] and [LA_out_] increased several-fold [Fig. 6A(c)], accompanied by increases in [GLUT1], [Glucose], [CA9], and [MCT] [Fig. 6B(c)]. The GLUT1-mediated increase in glucose uptake further activated the glycolytic pathway, responsible for massive accumulation of lactic acid; meanwhile, enhanced CA9 and MCT production facilitated LA_out_ accumulation. This could further increase the acid concentration in the microenvironment, and the tumor cell itself could become necrotic due to acidosis-activated BNIP3 caused by high [LA_in_].

The moderate increases in [LA_in_] and [LA_out_] evoked an acidic microenvironment under moderate hypoxia (see region II in Fig. 6C); by contrast, their remarkable increases resulted in acidosis under severe hypoxia (see region I). We further dissected the contribution of each regulator (including PFKL, GLUT1, MCT, and CA9) by setting their respective synthesis rates to 0 (control case); $\Delta_{{\mathrm{LA}}_{\text{in}}}$ and $\Delta_{{\mathrm{LA}}_{\text{out}}}$ separately denoted the changes in [LA_in_] and [LA_out_] relative to the control case (Fig. 6D). PFKL deficiency led to decreases in [LA_in_] and [LA_out_] across hypoxia, whereas GLUT1 deficiency led to their reduction only under severe hypoxia. This difference suggests that cells activate distinct pathways to enhance glycolysis and avoid high intracellular acidity when faced with different degrees of hypoxia (Fig. 6D). MCT acts as a generic scavenger across hypoxia; when its production was blocked, the decrease in [LA_in_] was always accompanied by an increase in [LA_out_]. By contrast, CA9 is a specific scavenger, and its deletion elicited an increase in [LA_in_] and a decrease in [LA_out_] mainly under severe hypoxia.

Under severe hypoxia, the competition between GLUT1 and CA9 affected the intracellular pH and BNIP3 activation. Both [LA_in_] and [LA_out_] rose with increasing $k_{\text{sGLUT}1}$ (production rate of GLUT1) [Fig. 6E(a) and (b)], while a profound LA_in_-to-LA_out_ shift occurred at larger $k_{\mathrm{sCA}9}$ (production rate of CA9) values. BNIP3 activation was modulated by both GLUT1 and CA9. At small or moderate $k_{\text{sGLUT}1}$, BNIP3^*^ accumulation was repressed due to the pumping of H^+^ by CA9; BNIP3^*^ was sufficiently activated at large $k_{\text{sGLUT}1}$ when excessive lactic acid accumulated in cells, leading to cell necroptosis [Fig. 6E(c)].

These results suggest that cells utilize several pathways to enhance glycolysis, thereby ensuring sufficient energy production and preventing acidosis. The accumulation of acid in internal or external environments depends on the severity of hypoxia and the selective expression of target genes of HIF-1 (Fig. 6F). Mildly hypoxic cells use HIF-1α-aOH to induce mild expression of PFKL, leading to weak accumulation of intracellular acid; meanwhile, moderately induced MCT exports small amounts of H^+^. Under moderate hypoxia, HIF-1α-aOH is markedly elevated, leading to enhanced PFKL expression and intracellular acid accumulation; coexpression of MCT and CA9 results in a large amount of H^+^ spillover, promoting the formation of acidic environment. Severely hypoxic cells further increase inward glucose transport by enhancing GLUT1 production, while elevated expression of CA9 and MCT further neutralizes intracellular H^+^. If CA9 fails to counteract the massive GLUT1-induced intracellular accumulation of H^+^, acidosis-dependent necroptosis will be triggered.

## Discussion and Conclusion

**Fig. 7.**
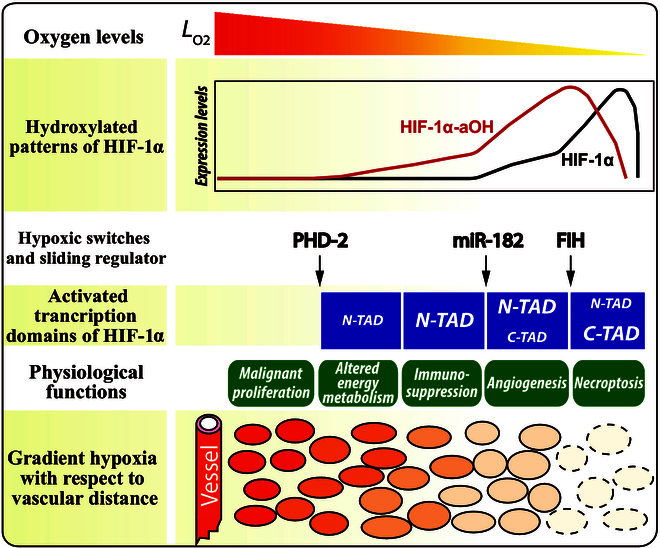
Schematic of the HIF-1α-mediated cellular response to graded hypoxia. The intensity of the filled colors in the bottom row is associated with the oxygen levels.

The hierarchical hypoxic microenvironment in solid tumors leads to heterogeneous responses to hypoxia in tumor cells, such as inducing an immune-resistant quiescent state in some cells and promoting tumor metastasis through the formation of highly permeable tumor blood vessels [6,68]. However, how tumor cells autonomously regulate their signaling networks to adapt to varying degrees of hypoxia remains an unresolved issue. Given that HIF-1 is a key regulator of the hypoxic response and produces typical morphological differences in tumor tissues, our study developed a network model to reveal how tumor cells utilize the HIF-1 signaling pathway to adapt to graded hypoxia. HIF-1 is progressively activated due to the modulation of its activity by oxygen level-dependent hydroxylases PHD-2/FIH and their regulators (e.g., miR-182); consequently, representative adaptive processes, including altered energy metabolism, immunosuppression, angiogenesis, and necroptosis, occur sequentially with worsening hypoxia when cells are distant from the blood vessels (Fig. 7). From a network perspective, the progressive induction of different responses depends on the selective activation of downstream signaling pathways, guided by the interconnected feedback loops involving HIF-1α, PHD-2, FIH, and miR-182, establishing an autonomous hypoxia-adaptive mechanism. This mechanism also orchestrates multiple aspects of anaerobic glycolysis including glucose absorption and the production and export of acid to ensure substantial ATP generation for cellular survival, while sustaining an acidic tumor microenvironment.

miRNAs can play a prominent role in the modulation of HIF-1-dependent cellular adaptation to hypoxia. miR-182 exemplifies a category of miRNAs that are induced by HIF-1 and subsequently modulate HIF-1 through the hydroxylases PHD-2 and FIH, thereby enclosing multiple feedback loops. As indicated in this work, miR-182 potentially acts as a modulator, facilitating VEGF induction (tumor angiogenesis) across defined hypoxic conditions [21]. miR-182 plays a role in a variety of processes, including proliferation, apoptosis, differentiation, glucose metabolism, invasion, and metastasis [20,69–71]. Moreover, a variety of factors including MINT3 [72], PKD-1 [17], miR-351 [73], and miR-210 [74] are also involved in HIF-1 regulation by targeting PHD-2 and FIH. It is intriguing to investigate how miR-182 synergizes with other enzymatic regulators to modulate HIF-1 signaling.

Considering the feedback loops involving HIF-1α, miR-182, PHD-2, and FIH, we underscore the crucial role of miR-182 in regulating VEGF in response to hypoxia. Indeed, other HIF-1α-induced miRNAs also play important roles in the hypoxic response [75]. For instance, HIF-1α induces miR-155 to relieve its activity during prolonged hypoxia [19], while miR-122 enhances hepatic ischemia tolerance by promoting hepatic protection through a feedback mechanism that enhances PHD-1 inhibition [76]. Moreover, HIF-2α plays a remarkable role in hypoxic response by transactivating some miRNAs. For example, HIF-2α induces the expression of miR-29a in CD4^+^ cells, which is crucial for alleviating TH1-mediated inflammatory responses [77]. In the future, including more miRNAs in the network model of the hypoxic response may provide valuable insights for the precise treatment of various diseases.

Sufficient activation of BNIP3 is responsible for hypoxic necroptosis, in which BNIP3 dimerizes and inserts into the mitochondrial membrane to regulate its permeability [78,79]. Although BNIP3 is expressed at various hypoxia levels, it induces cell necroptosis only under severe hypoxia, establishing a scenario in which the expression and activation of BNIP3 are separately regulated [27,45]. This could result from the difficulty in inducing acidosis under mild hypoxia when lactic acid production is negligible. In the absence of acidosis, BNIP3 is unstable and exhibits low affinity to mitochondria [28]. Alternatively, only when BNIP3 accumulates sufficiently can it counteract the formation of heterodimers with Bcl-2, Bcl-xL, or Bax; indeed, the expression of BNIP3 in severe hypoxia is nearly twice that observed in mild hypoxia [27,28,80]. In addition, BNIP3 could also promote cell survival through autophagy, which emerges under moderate hypoxia [81].

The role of necroptosis in tumor progression is worth discussing. As an essential hallmark of aggressive tumors, necroptosis promotes cell survival by amplifying HIF-1α signaling as well as activating the NF-κB and PI3K/mTOR pathways [82]. Consistently, necrotic extracts from dying cells promote migration and invasion of glioblastoma cancer stem cells with reduced oxygen tension [83]. Necrotic lysates enhance endothelial cell proliferation and angiogenesis, promoting cell invasion and migration [84]. Thus, necroptosis may be a strategy for tumor progression, and the necroptosis of individual cells facilitates more robust survival of the tumor cell community. Considering the role of necrotic lysates in promoting tumor vascularization, a cross-link between VEGF and necrotic downstream pathways could be identified [82]. It would be interesting to take into account the inflammatory regulatory pathways to clarify the mechanisms underlying the coordination of necroptosis, inflammation, and hypoxia in tumor progression [83,85–87]. Following VEGF-mediated neovascularization, HIF-1 signaling is attenuated with elevated oxygen pressure, creating a long-range feedback loop between HIF-1, VEGF, and hypoxia detection. Integrating this loop into our model is challenging but intriguing since a considerable portion of tumor cells are subjected to at least 2 cycles of hypoxia [88].

Our results suggest that cell necroptosis could be avoided by disrupting the expression and activation of BNIP3 or HIF-1 under severe hypoxia. FIH inhibitors could be exploited to destabilize HIF-1α since FIH contributes to the stabilization of HIF-1α under hypoxia [37]. Alternatively, p53 may be a candidate for inhibiting necroptosis for the following reasons: (a) p53 directly inhibits BNIP3 expression [89]; (b) p53 reduces HIF-1 activity by competing for p300 and induces Mdm2 production to degrade HIF-1 [90,91]; and (c) multiple targets of p53 are associated with the inhibition of glycolysis, which disrupts acidosis and BNIP3 activation [92]. Probing the interplay between p53 and HIF-1 revealed a mechanism for activating p53 under severe hypoxia [93]. Replacing BNIP3-mediated necroptosis with p53-mediated apoptosis may be a viable approach for tumor suppression.

Our findings could be validated by designing experiments specifically. Based on the experiment conducted by Dayan et al. [27], devising experiments that encompass HIF-1 target genes within our model across varying oxygen concentrations could provide validation for our findings regarding the relationship between target gene expression and the degree of hypoxia. To further investigate the role of miR-182 in regulating VEGF expression under hypoxia, it is advisable to knock out miR-182 and assess VEGF expression under different oxygen levels. In addition, the effects of oxygen levels on the expression and activation of BNIP3 could be tested indirectly by assessing the key downstream components involved in the BNIP3-mediated necroptosis pathway, such as RIPK1, RIPK3, and MLKL [78,79]. Inhibiting these factors to promote cell survival could clarify whether BNIP3 activity is markedly enhanced under severe hypoxia.

Our work could provide insight into the mechanism underlying tumor cell adaptation to hypoxia, but some limitations still exist. First, a critical assessment of the model’s simplifications against real biological processes is essential. For example, we did not include the neuronal guidance protein Netrin-1, which is a noteworthy target of HIF-1 [53]. Incorporating Netrin-1 into the model could enhance the comprehension of the regulatory details of immunosuppression and energy metabolism. Second, we omitted another HIF, HIF-2α, which performs different functions compared to HIF-1α. It has been reported that HIF-1α and HIF-2α have distinct transcriptional targets: the former primarily induces genes involved in glycolysis in response to acute hypoxia, whereas the latter regulates genes related to erythropoietin under chronic hypoxia [94]. Moreover, they also cooperatively induce genes such as VEGF and GLUT-1 [94]. Incorporating HIF-2α into the model would contribute to understanding how cells utilize HIF pathways to adapt to hypoxia. Third, the crosstalk between HIF-1 and NF-κB is not included in our model [95]. Their interaction is frequently observed in the hypoxic response. For example, NF-κB can act as a transactivator of HIF-1α [96]. They also cooperate to induce genes such as interleukin-6, cyclooxygenase-2, and matrix metalloproteinase-9 [95]. Including NF-κB in the model could help elucidate how hypoxia triggers chronic inflammation through the HIF-1α-NF-κB interaction. In addition, biological noise is not considered in the present model [97]. Kang et al. [98] proposed a theoretical framework for the stochastic dynamics of the HIF-1 network based on landscape topography. Incorporating their scheme into our research could provide valuable insights into the significance of stochasticity in cellular adaptation to hypoxia.

Our results may provide clues for treating hypoxia-associated diseases. Therapeutic strategies targeting HIF can be classified as HIF stabilizers and inhibitors tailored in different diseases [99]. HIF stabilizers up-regulate the α subunit of HIFs by inhibiting PHDs, primarily used to treat anemia caused by hypoxia [100,101]. In contrast, HIF inhibitors block the dimerization of HIF subunits, mainly used in cancer therapy to inhibit tumor growth in clear cell renal carcinoma [102]. However, in patients with both ischemic cardiovascular disease and cancer, HIF inhibitors may interfere with blood flow restoration, potentially exacerbating tissue damage [103]. Given the selective expression of genes targeted by HIF-1α, optimal treatment strategies could be developed by concurrently targeting multiple factors and accounting for interpatient variability. The proposed model elucidates how inhibition of HIF-1 regulatory factors, including hydroxylases and miR-182, selectively attenuates distinct hypoxic responses, such as angiogenesis and immunosuppression.

## Methods

Based on the typical morphological features observed in tumor tissue at varying distances from blood vessels, glycolysis, immunosuppression, angiogenesis, and necroptosis were selected as 4 representative processes in response to hypoxia [3]. HIF-1 has been shown to transcriptionally regulate several enzymes involved in the induction of glycolysis [24]. For simplicity, PFKL was selected as a marker for the transition from oxidative metabolism to glycolysis [27]. Considering the role of BNIP3 in necroptosis and the role of VEGF in angiogenesis at the periphery of tumor necrotic regions, VEGF and BNIP3 were chosen as markers for angiogenesis and necroptosis, respectively [3,28]. In addition, miR-182 and the pH-regulating factors MCT and CA9 were also considered in the regulation of these processes [21,45].

Besides the input (hypoxia) and 4 outputs (glycolysis, immunosuppression, angiogenesis, and necroptosis), the model consists of 31 nodes and is mathematically characterized by 28 ordinary differential equations (ODEs). The concentration of each species (denoted as [...]) is described by the state variables in the ODEs, with their detailed definitions provided in Supplementary Method S1. In the model, the enzyme-catalyzed reactions are characterized by Michaelis-Menten kinetics while HIF-1α-dependent transcription of the target genes is described by the Hill functions. In addition, protein degradation follows the law of mass action. The definition and initial values of each variable and parameter are listed in Tables S1 and S2, respectively. Of note, besides the parameters based on experimental results, other parameters in the model are estimated through trial and error to ensure that the simulation results are consistent with experimental observations (see Table S2 for details). Moreover, a sensitivity analysis was performed to ensure the model’s robustness to perturbations in parameter values (as shown in Fig. 4D). Oscill8 was used to solve the ODEs and perform single- and 2-parameter bifurcation analysis. The value of oxygen level *L*_O2_ corresponds to the percentage of oxygen volume in the air. The initial value of each variable was set to its steady-state value at 21% O_2_. Time is in units of minutes.

## Data Availability

All data needed to evaluate the conclusions are presented in the paper and the Supplementary Materials. Custom codes will be available upon request to X.-P.Z. (Email: zhangxp@nju.edu.cn).

## Supplementary Materials

Supplementary 1 Supplemental Method S1 Tables S1 and S2
