# Supplementary material for: Progressive Deactivation of Hydroxylases Controls Hypoxia-Inducible Factor-1α-Coordinated Cellular Adaptation to Graded Hypoxia
Source: Research (Wash D C). 2025 Apr 1;8:0651. doi: 10.34133/research.0651 (PMC11960303; doi:10.34133/research.0651)
Supplement: Supplementary 1 — Supplemental Method S1 Tables S1 and S2 [file research.0651.f1.pdf]

Supplementary materials

Progressive deactivation of hydroxylases controls hypoxia-inducible factor-1α-coordinated cellular adaptation to graded hypoxia

In this study, we developed a network model to highlight the critical role of HIF1α hydroxylases in guiding cellular decision-making and enabling optimal adaptation to hypoxia. The ordinary differential equations, along with their initial values and parameters, are provided in this document. Notably, the initial values correspond to the steady-state levels of each variable under 21% O_2.

Supplemental Method S1：Equations of the model

$$\frac{d[\text{HIF-1}\alpha]}{dt} = k_{\text{sHIF1a}} - k_{\text{deHIF1a1}}[\text{FIH}^*]\frac{[\text{HIF-1}\alpha]}{[\text{HIF-1}\alpha]+j_{\text{FIH}}} - k_{\text{deHIF1a2}}[\text{PHD-2}^*]\frac{[\text{HIF-1}\alpha]}{[\text{HIF-1}\alpha]+j_{\text{PHD}}} - d_{\text{HIF1a}}[\text{HIF-1}\alpha] - k_{\text{deHIF1a3}}\frac{[\text{HIF-1}\alpha]}{[\text{FIH}^*]+j_{\text{FIHa}}} \quad (1)$$

$$\frac{d[\text{HIF1}\alpha\text{-aOH}]}{dt} = k_{\text{sHIF1aa}} + k_{\text{deHIF1a1}}[\text{FIH}^*]\frac{[\text{HIF-1}\alpha]}{[\text{HIF-1}\alpha]+j_{\text{FIH}}} - k_{\text{deHIF1aa}}[\text{PHD-2}^*]\frac{[\text{HIF-1}\alpha\text{-aOH}]}{[\text{HIF-1}\alpha\text{-aOH}]+j_{\text{PHD}}} - d_{\text{HIF1aa}}[\text{HIF-1}\alpha\text{-aOH}] \quad (2)$$

$$\frac{d[\text{miR-182}]}{dt} = k_{\text{smiR1820}} + k_{\text{smiR1821}}\frac{[\text{HIF1}\alpha\text{-aOH}]^4}{[\text{HIF1}\alpha]^4+{j_{\text{smiR1821}}}^4} + k_{\text{smiR1822}}\frac{[\text{HIF-1}\alpha]^4}{[\text{HIF-1}\alpha]^4+{j_{\text{smiR1822}}}^4} - d_{\text{miR182}}[\text{miR-182}] \quad (3)$$

$$\frac{d[\text{PHD-2}_\text{T}]}{dt} = k_{\text{sPHDT0}} + \left(k_{\text{sPHDT1}}\frac{[\text{HIF-1}\alpha]^4}{[\text{HIF-1}\alpha]^4+{j_{\text{sPHDT1}}}^4} + k_{\text{sPHDT2}}\frac{[\text{HIF-1}\alpha\text{-aOH}]^4}{[\text{HIF1}\alpha\text{-aOH}]^4+{j_{\text{sPHDT2}}}^4}\right)\frac{j_{miR1821}^4}{[miR-182]^4+j_{miR1821}^4} - d_{\text{PHD2T}}[\text{PHD-2}_\text{T}] \quad (4)$$

$$\frac{d[\text{PHD-2}^*]}{dt} = k_{\text{acPHD}}\frac{O_2}{O_2+\frac{j_{\text{O2PHD}}}{a}}[\text{PHD-2}] - k_{\text{dePHDa}}[\text{PHD-2}^*] \quad (5)$$

$$[\text{PHD-2}] = [\text{PHD-2}_\text{T}] - [\text{PHD-2}^*] \quad (6)$$

$$\frac{d[\text{FIH}_\text{T}]}{dt} = k_{\text{sFIHT0}} + k_{\text{sFIHT}}\frac{j_{miR1822}^4}{[miR-182]^4+j_{miR1822}^4} - d_{\text{FIHT}}[\text{FIH}_\text{T}] \quad (7)$$

$$\frac{d[\text{FIH}^*]}{dt} = k_{\text{acFIH}}\frac{O_2}{O_2+\frac{j_{\text{O2FIH}}}{a}}\frac{[\text{FIH}]}{[\text{FIH}]+j_{\text{acFIH}}} - k_{\text{deFIHa}}\frac{[\text{FIH}^*]}{[\text{FIH}^*]+j_{\text{deFIHa}}} \quad (8)$$

$$[\text{FIH}] = [\text{FIH}_\text{T}] - [\text{FIH}^*] \quad (9)$$

$$\frac{d[\text{PFKL}]}{dt} = k_{\text{sPFKL0}} + k_{\text{sPFKL}}\frac{[\text{HIF1}\alpha\text{-aOH}]^4}{[\text{HIF1}\alpha\text{-aOH}]^4+{j_{\text{sPFKL}}}^4} - d_{\text{PFKL}}[\text{PFKL}] - k_{\text{acPFKL}}\frac{[\text{PFKL}]}{[\text{PFKL}]+j_{\text{acPFKL}}} + k_{\text{dePFKLa}}\frac{[\text{Lactic acid}]_{\text{in}}}{[\text{Lactic acid}]_{\text{in}}+j_{\text{LA1}}}\frac{[\text{PFKL}^*]}{[\text{PFKL}^*]+j_{\text{dePFKLa}}} \quad (10)$$

$$\frac{d[\text{PFKL}^*]}{dt} = k_{\text{acPFKL}}\frac{[\text{PFKL}]}{[\text{PFKL}]+j_{\text{acPFKL}}} - k_{\text{dePFKLa}}\frac{[\text{Lactic acid}]_{\text{in}}}{[\text{Lactic acid}]_{\text{in}}+j_{\text{LA1}}}\frac{[\text{PFKL}^*]}{[\text{PFKL}^*]+j_{\text{dePFKLa}}} - d_{\text{PFKLa}}[\text{PFKL}^*] \quad (11)$$

$$\frac{d[\text{VEGF}]}{dt} = k_{\text{sVEGF0}} + k_{\text{sVEGF}}\frac{[\text{HIF-1}\alpha]^4}{[\text{HIF-1}\alpha]^4+{j_{\text{sVEGF}}}^4} - d_{\text{VEGF}}[\text{VEGF}] \quad (12)$$

$$\frac{d[\text{CA9}]}{dt} = k_{\text{sCA90}} + k_{\text{sCA9}}\frac{[\text{HIF-1}\alpha]^4}{[\text{HIF-1}\alpha]^4+{j_{\text{sCA9}}}^4} - d_{\text{CA9}}[\text{CA9}] \quad (13)$$

$$\frac{d[\text{MCT}]}{dt} = k_{\text{sMCT0}} + k_{\text{sMCT1}}\frac{[\text{HIF-1}\alpha\text{-aoH}]^4}{[\text{HIF-1}\alpha\text{-aoH}]^4+{j_{\text{sMCT1}}}^4} + k_{\text{sMCT2}}\frac{[\text{HIF-1}\alpha]^4}{[\text{HIF1}\alpha]^4+{j_{\text{sMCT2}}}^4} - d_{\text{MCT}}[\text{MCT}] \quad (14)$$

$$\frac{d[\text{BNIP3}]}{dt} = k_{\text{sBNIP30}} + k_{\text{sBNIP31}}\frac{[\text{HIF-1}\alpha\text{-aOH}]^4}{[\text{HIF-1}\alpha\text{-aOH}]^4+{j_{\text{sBNIP31}}}^4} + k_{\text{sBNIP32}}\frac{[\text{HIF-1}\alpha]^4}{[\text{HIF-1}\alpha]^4+{j_{\text{sBNIP32}}}^4} - d_{\text{BNIP3}}[\text{BNIP3}] - k_{\text{acBNIP3}}\frac{[\text{Lactic acid}_{\text{in}}]}{[\text{Lactic acid}_{\text{in}}]+j_{\text{LA2}}}\frac{[\text{BNIP3}]}{[\text{BNIP3}]+j_{\text{acBNP3}}} + k_{\text{deBNIP3}}\frac{[\text{BNIP3}^*]}{[\text{BNIP3}^*]+j_{\text{deBNIP3a}}} \quad (15)$$

$$\frac{d[\mathrm{BNIP3^*}]}{dt} = k_{\mathrm{acBNIP3}} \frac{[\text{Lactic acid}_{\mathrm{in}}]}{[\text{Lactic acid}_{\mathrm{in}}]+j_{\mathrm{LA2}}} \frac{[\mathrm{BNIP3}]}{[\mathrm{BNIP3}]+j_{\mathrm{acBNP3}}} - k_{\mathrm{deBNIP3}} \frac{[\mathrm{BNIP3^*}]}{[\mathrm{BNIP3^*}]+j_{\mathrm{deBNIP3a}}} - d_{\mathrm{BNIP3a}}[\mathrm{BNIP3^*}] \quad (16)$$

$$\frac{d[\text{GLUT-1}]}{dt} = k_{\mathrm{sGLUT10}} + k_{\mathrm{sGLUT1}} \frac{[\text{HIF-1}\alpha]^4}{[\text{HIF-1}\alpha]^4+j_{\mathrm{sGLUT1}}{}^4} - d_{\mathrm{GLUT1}}[\text{GLUT-1}] \quad (17)$$

$$\frac{d[\mathrm{Glucose_{in}}]}{dt} = (k_{\mathrm{strans1}} + k_{\mathrm{strans2}}[\text{GLUT-1}])Glucose_{\mathrm{out}} - (k_{\mathrm{tP0}} + k_{\mathrm{tP}} \frac{[\mathrm{PFKL^*}]}{[\mathrm{PFKL^*}]+j_{\mathrm{PFKLa}}})[\mathrm{Glucose_{in}}] - d_{\mathrm{Glucose}}[\mathrm{Glucose_{in}}] \quad (18)$$

$$\frac{d[\mathrm{Pyruvate}]}{dt} = (k_{\mathrm{tP0}} + k_{\mathrm{tP}} \frac{[\mathrm{PFKL^*}]}{[\mathrm{PFKL^*}]+j_{\mathrm{PFKLa}}})[\mathrm{Glucose_{in}}] - k_{\mathrm{tPL}} \frac{j_{\mathrm{corO2}}}{\mathrm{O_2}*a+j_{\mathrm{corO2}}}[\mathrm{Pyruvate}] - k_{\mathrm{tPAC}} \frac{\mathrm{O_2}*a}{\mathrm{O_2}*a+j_{\mathrm{corO2}}}[\mathrm{Pyruvate}] - d_{\mathrm{Pyruvate}}[\mathrm{Pyruvate}] \quad (19)$$

$$\frac{d[\mathrm{ACoA}]}{dt} = k_{\mathrm{tPAC}} \frac{\mathrm{O_2}*a}{\mathrm{O_2}*a+j_{\mathrm{corO2}}}[\mathrm{Pyruvate}] - d_{\mathrm{ACoA}}[\mathrm{ACoA}] \quad (20)$$

$$\frac{d[\text{Lactic acid}_{\mathrm{in}}]}{dt} = k_{\mathrm{tPAC}} \frac{\mathrm{O_2}*a}{\mathrm{O_2}*a+j_{\mathrm{corO2}}}[\mathrm{Pyruvate}] - (k_{\mathrm{outLA1}}[\mathrm{CA9}] + k_{\mathrm{outLA2}}[\mathrm{MCT}])[\text{Lactic acid}_{\mathrm{in}}] + k_{\mathrm{inLA}}[\text{Lactic acid}_{\mathrm{out}}] - d_{\mathrm{LAin}}[\text{Lactic acid}_{\mathrm{in}}] \quad (21)$$

$$\frac{d[\text{Lactic acid}_{\mathrm{out}}]}{dt} = (k_{\mathrm{outLA1}}[\mathrm{CA9}] + k_{\mathrm{outLA2}}[\mathrm{MCT}])[\text{Lactic acid}_{\mathrm{in}}] - k_{\mathrm{inLA}}[\text{Lactic acid}_{\mathrm{out}}] - d_{\mathrm{LAout}}[\text{Lactic acid}_{\mathrm{out}}] \quad (22)$$

$$\frac{\mathrm{d}[\mathrm{Cx43}]}{\mathrm{dt}} = k_{\mathrm{sCx430}} + k_{\mathrm{sCx431}} \frac{[\text{HIF-1}\alpha\text{-aOH}]^4}{[\text{HIF-1}\alpha\text{-aOH}]^4+j_{\mathrm{sC431}}{}^4} + k_{\mathrm{sCx432}} \frac{[\text{HIF-1}\alpha]^4}{[\text{HIF-1}\alpha]^4+j_{\mathrm{sCx432}}{}^4} - d_{\mathrm{Cx43}}[\mathrm{Cx43}] \quad (23)$$

$$\frac{d[\mathrm{ATP}]}{dt} = (k_1[\mathrm{Pyruvate}] + k_2[\mathrm{ACoA}])[\mathrm{ADP}] - d_{\mathrm{ATP}}[\mathrm{ATP}] + d_{\mathrm{ADP}}[\mathrm{ADP}]^2 - d_{\mathrm{AMP}}[\mathrm{AMP}][\mathrm{ATP}] - k_{\mathrm{outATP}}[\mathrm{Cx43}][\mathrm{ATP}] \quad (24)$$

$$\frac{d[\mathrm{AMP}]}{dt} = d_{\mathrm{ADP}}[ADP]^2 - d_{\mathrm{AMP}}[\mathrm{AMP}][\mathrm{ATP}] \quad (25)$$

$$[\mathrm{ADP}] = [\mathrm{AXP}_t] - [\mathrm{ATP}] - [\mathrm{AMP}] \quad (26)$$

$$\frac{d[\mathrm{ATP_{out}}]}{dt} = k_{\mathrm{outATP}}[\mathrm{Cx43}][\mathrm{ATP}] - d_{\mathrm{ATPout}}[\mathrm{ATP_{out}}] - k_{\mathrm{tsATPout}}[\mathrm{CD73}][\mathrm{ATP_{out}}] \quad (27)$$

$$\frac{d[\mathrm{CD73}]}{dt} = k_{\mathrm{sCD730}} + k_{\mathrm{sCD731}} \frac{[\mathrm{HIF}-1\alpha-a\mathrm{OH}]^4}{[\mathrm{HIF}-1\alpha-a\mathrm{OH}]^4+j_{\mathrm{sCD731}}{}^4} + k_{\mathrm{sCD732}} \frac{[\mathrm{HIF}-1\alpha]^4}{[\mathrm{HIF}-1\alpha]^4+j_{\mathrm{sCD732}}{}^4} - d_{\mathrm{CD73}}[\mathrm{CD73}] \quad (28)$$

$$\frac{d[\mathrm{Adenosine}]}{dt} = k_{\mathrm{tsATPout}}[\mathrm{CD73}][\mathrm{ATP_{out}}] - d_{\mathrm{Adenosine}}[\mathrm{Adenosine}] \quad (29)$$

$$\frac{d[\mathrm{A2B}]}{dt} = k_{\mathrm{sA2B0}} + k_{\mathrm{sA2B1}} \frac{[\mathrm{HIF}-1\alpha-a\mathrm{OH}]^4}{[\mathrm{HIF}-1\alpha-a\mathrm{OH}]^4+j_{\mathrm{sA2B1}}{}^4} + \mathrm{j}_{\mathrm{sA2B2}} \frac{[\mathrm{HIF}-1\alpha]^4}{[\mathrm{HIF}-1\alpha]^4+j_{\mathrm{sA2B2}}{}^4} - d_{\mathrm{A2B}}[\mathrm{A2B}] \quad (30)$$

$$\frac{d[\mathrm{A2B^*}]}{dt} = k_{\mathrm{acA2B}} \frac{[\mathrm{Adenosine}]}{[\mathrm{Adenosine}]+j_{\mathrm{Adenosine}}} \frac{[\mathrm{A2B}]}{[\mathrm{A2B}]+j_{\mathrm{acA2B}}} - k_{\mathrm{deA2Ba}} \frac{[\mathrm{A2B^*}]}{[\mathrm{A2B^*}]+j_{\mathrm{deA2Ba}}} \quad (31)$$

SUPPLEMENTAL TABLE S1: DESCRIPTION AND INITIAL VALUES OF VARIABLES

Variable	Description	Initial value
[HIF-1α]	Concentration of HIF-1α	0.19791
[HIF-1α-aOH]	Concentration of HIF-1α-aOH	0.50826
[miR-182]	Concentration of miR-182	0.50053
[PHD-2_T]	Total concentration of PHD-2 and PHD-2*	9.83889
[PHD-2*]	Concentration of active PHD-2	3.06758
[FIH_T]	Total concentration of FIH and FIH*	10.099
[FIH*]	Concentration of active FIH	10.09094
[PFKL]	Concentration of PFKL	0.03281
[PFKL*]	Concentration of active PFKL*	0.06553
[VEGF]	Concentration of VEGF	0.04121
[CA9]	Concentration of CA9	0.02148
[MCT]	Concentration of MCT	0.0285
[BNIP3]	Concentration of BNIP3	0.05165
[BNIP3*]	Concentration of active BNIP3	4.10744E-4
[GLUT-1]	Concentration of GLUT-1	0.10002
[$Glucose_{in}$]	Concentration of intracellular Glucose	3.79915
[Pyruvate]	Concentration of Pyruvate	0.74558
[ACoA]	Concentration of Acetyl-CoA	0.6799
[Lactic $acid_{in}$]	Concentration of intracellular Lactic acid	0.63555
[Lactic $acid_{out}$]	Concentration of extracellular Lactic acid	0.01059
[Cx43]	Concentration of Cx43	0.04021
[ATP]	Concentration of intracellular ATP	7.1201
[AMP]	Concentration of AMP	0.80568
[ATP_{out}]	Concentration of extracellular ATP	0.08519
[CD73]	Concentration of CD73	0.04104
[Adenosine]	Concentration of Adenosine	0.0035
[A2B]	Concentration of A2B	0.04105
[A2B*]	Concentration of active A2B	4.21639E-5

SUPPLMENTAL TABLE S2: PARAMETERS OF THE MODEL

Variable	Description	Value	Reference
O_2%	Volume percentage of O_2 in air	[0% , 21%]	
a	Value of O_2 level in μM corresponds to 1% O_2	9.86	[1]
j_{O2PHD}	Threshold of O_2 for PHD-2 activation	250	[2]
j_{O2FIH}	Threshold of O_2 for FIH activation	90	[2]
k_{sHIF1a}	Production rate of HIF-1α	0.4 min^{-1}	[3]
$k_{deHIF1a1}$	FIH*-dependent hydroxylation rate of HIF-1α	0.11 min^{-1}	Assumed
j_{FIH}	Michaelis constant for HIF-1α as a substrate of FIH*	0.5	Assumed
$k_{deHIF1a2}$	PHD-2*-dependent hydroxylation rate of HIF-1α	0.3 min^{-1}	Assumed
j_{PHD}	Michaelis constant for HIF-1α as a substrate of PHD-2*	2	[3]
d_{HIF1a}	Basal degradation rate of HIF-1α	0.01	[4]
$k_{deHIF1a3}$	PHD-2-independent degradation rate of HIF-1α	0.02 min^{-1}	Assumed
j_{FIHa}	Threshold of FIH* required for inhibiting the PHD-2-independent degradation of HIF-1α	0.01	Assumed
$k_{sHIF1aa}$	Basal production rate of HIF-1α-aOH	0.0001 min^{-1}	Assumed
$k_{deHIF1aa}$	PHD-2*-dependent hydroxylation rate of HIF-1α-aOH	0.5 min^{-1}	Assumed
d_{HIF1aa}	Degradation rate of HIF-1α-aOH	0.008 min^{-1}	Assumed
$k_{smiR1820}$	Basal production rate of miR-182	0.001 min^{-1}	Assumed
$k_{smiR1821}$	HIF-1α-aOH-dependent production rate of miR-182	0.009 min^{-1}	Assumed
$j_{smiR1821}$	Michaelis constant of HIF-1α-aOH-dependent production of miR-182	5	Assumed
$k_{sminR1822}$	HIF-1α-dependent production rate of miR-182	0.018 min^{-1}	Assumed
$j_{smiR1822}$	Michaelis constant of HIF-1α-dependent production of miR-182	4	Assumed
d_{miR182}	Degradation rate of miR-182	0.002 min^{-1}	Assumed
k_{sPHDT0}	Basal production rate of PHD-2_T	0.0001 min^{-1}	Assumed
k_{sPHDT1}	HIF-1α-dependent production rate of PHD-2_T	0.02 min^{-1}	[5]
j_{sPHDT1}	Michaelis constant of HIF-1α-dependent PHD-2_T production	0.1	Assumed
k_{sPHDT2}	HIF-1α-aOH-dependent production rate of PHD-2_T	0.03 min^{-1}	Assumed
j_{sPHDT2}	Michaelis constant of HIF-1α-aOH-dependent PHD-2_T production	0.9	Assumed
$j_{miR1821}$	Threshold of miR-182-dependent inhibition of PHD production	10	Assumed
d_{PHDT}	Basal degradation rate of PHD-2_T	0.0022 min^{-1}	Assumed
k_{acPHD}	Activation rate of PHD-2	1 min^{-1}	Assumed
k_{dePHDa}	Inactivation rate of PHD-2*	1 min^{-1}	[3]
k_{sFIHT0}	Basal production rate of FIH_T	0.001 min^{-1}	Assumed
k_{sFIHT}	miR-182 inhibited production rate of FIH_T	0.1 min^{-1}	Assumed
$j_{miR1822}$	Threshold of miR-182-dependent inhibition of FIH production	5	Assumed
d_{FIHT}	Basal degradation rate of FIH_T	0.01 min^{-1}	Assumed
k_{acFIH}	Activation rate of FIH	7 min^{-1}	Assumed
j_{acFIH}	Michaelis constant of FIH activation	0.1	Assumed
k_{deFIHa}	Inactivation rate of FIH*	0.4 min^{-1}	Assumed
j_{deFIHa}	Michaelis constant of FIH* inactivation	1	Assumed
k_{sPFKL0}	Basal production rate of PFKL	0.0004 min^{-1}	Assumed
k_{sPFKL}	HIF-1α-aOH-dependent production rate of PFKL	0.03 min^{-1}	Assumed
j_{sPFKL}	Michaelis constant of HIF-1α-aOH -dependent PFKL production	2	Assumed
d_{PFKL}	Degradation rate of PFKL	0.008 min^{-1}	Assumed
k_{acPFKL}	Activation rate of PFKL	0.2 min^{-1}	Assumed
j_{acPFKL}	Michaelis constant of activation rate of PFKL	2	Assumed
$k_{dePFKLa}$	Lactic $acid_{in}$-dependent inactivation rate of PFKL*	0.2 min^{-1}	Assumed
j_{LA1}	Threshold of Lactic $acid_{in}$ required for inhibiting PFKL activation	2	Assumed
$j_{dePFKLa}$	Michaelis constant of Lactic $acid_{in}$-dependent inactivation rate of PFKL*	1	Assumed
d_{PFKLa}	Degradation rate of PFKL*	0.004 min^{-1}	Assumed
k_{sVEGF0}	Basal production rate of VEGF	0.0002 min^{-1}	Assumed
k_{sVEGF}	HIF-1α-dependent production rate of VEGF	0.02 min^{-1}	Assumed
j_{sVEGF}	Michaelis constant of HIF-1α-dependent production of VEGF	1.5	Assumed
d_{VEGF}	Degradation rate of VEGF	0.005 min^{-1}	[6]
k_{sCA90}	Basal production rate of CA9	0.0001 min^{-1}	Assumed
k_{sCA9}	HIF-1α-dependent production rate of CA9	0.01 min^{-1}	Assumed
j_{sCA9}	Michaelis constant of HIF-1α-dependent production of CA9	1.2	Assumed
d_{CA9}	Degradation rate of CA9	0.005 min^{-1}	Assumed
k_{sMCT0}	Basal production rate of MCT	0.0001 min^{-1}	Assumed

SUPPLEMENTAL TABLE S2-CONTINUED

Variable	Description	Value	Reference
k_{sMCT1}	HIF-1α-aOH-dependent production rate of MCT	0.01 min^{-1}	Assumed
j_{sMCT1}	Michaelis constant of HIF-1α-aOH-dependent production of MCT	2	Assumed
k_{sMCT2}	HIF-1α-dependent production rate of MCT	0.01 min^{-1}	Assumed
j_{sMCT2}	Michaelis constant of HIF-1α-dependent production of MCT	2	Assumed
d_{MCT}	Degradation rate of MCT	0.005 min^{-1}	Assumed
$k_{sBNIP30}$	Basal production rate of BNIP3	0.0002 min^{-1}	Assumed
$k_{sBNIP31}$	HIF-1α-aOH-dependent production rate of BNIP3	0.01 min^{-1}	Assumed
$j_{sBNIP31}$	Michaelis constant of HIF-1α-aOH-dependent production of BNIP3	3	Assumed
$k_{sBNIP32}$	HIF-1α-dependent production rate of BNIP3	0.04 min^{-1}	Assumed
$j_{sBNIP32}$	Michaelis constant of HIF-1α-dependent production of BNIP3	8	Assumed
d_{BNIP3}	Degradation rate of BNIP3	0.004 min^{-1}	[7]
$k_{acBNIP3}$	Activation rate of BNIP3	2 min^{-1}	Assumed
j_{LA2}	Threshold of Lactic $acid_{in}$ required for promoting BNIP3 activation	6	Assumed
$j_{acBNIP3}$	Michaelis constant of BNIP3 activation	8	Assumed
$k_{deBNIP3a}$	Inactivation rate of BNIP3*	0.3 min^{-1}	Assumed
$j_{deBNIP3a}$	Michaelis constant of BNIP3* inactivation	0.1	Assumed
d_{BNIP3a}	Degradation rate of BNIP3*	0.004 min^{-1}	Assumed
$k_{sGLUT10}$	Basal production rate of GLUT-1	0.0005 min^{-1}	Assumed
k_{sGLUT1}	HIF-1α-dependent production rate of GLUT-1	0.02 min^{-1}	Assumed
j_{sGLUT1}	Michaelis constant of HIF-1α-dependent production of GLUT-1	4	Assumed
d_{GLUT1}	Degradation rate of GLUT-1	0.005 min^{-1}	Assumed
$Glucose_{out}$	Concentration of extracellular Glucose	10	Assumed
k_{trans1}	Transportation rate of glucose	0.1 min^{-1}	Assumed
k_{trans2}	GLUT-1-dependent transportation rate of glucose	0.2 min^{-1}	Assumed
k_{tP0}	Conversion rate of Glucose to Pyruvate	0.1 min^{-1}	Assumed
k_{tP}	PFKL*-dependent conversion rate of Glucose to Pyruvate	1 min^{-1}	Assumed
j_{PFKLa}	Threshold of PFKL* required for the conversion of Glucose to Pyruvate	0.5	Assumed
$d_{Glucosein}$	Degradation rate of $Glucose_{in}$	0.1 min^{-1}	Assumed
k_{tPL}	Conversion rate of Pyruvate to Lactic $acid_{in}$	0.1 min^{-1}	Assumed
j_{corO2}	O_2 threshold for the conversion of Pyruvate to Lactic $acid_{in}$	20	Assumed
k_{tPAC}	Conversion rate of Pyruvate to Acetyl-CoA	0.1 min^{-1}	Assumed
$d_{Pyruvate}$	Degradation rate of Pyruvate	1 min^{-1}	Assumed
d_{ACoA}	Degradation rate of Acetyl-CoA	0.1 min^{-1}	Assumed
k_{inLA}	Basal in-flow rate of Lactic $acid_{out}$	0.01 min^{-1}	Assumed
k_{outLA1}	CA9-dependent out-flow rate of Lactic $acid_{in}$	0.01min^{-1}	Assumed
k_{outLA2}	MCT-dependent out-flow rate of Lactic $acid_{in}$	0.01 min^{-1}	Assumed
d_{LAin}	Degradation rate of Lactic $acid_{in}$	0.01 min^{-1}	Assumed
d_{LAout}	Degradation rate of Lactic $acid_{out}$	0.02 min^{-1}	Assumed
k_{sCx430}	Basal production rate of CX43	0.0002 min^{-1}	Assumed
k_{sCx431}	HIF-1α-aOH-dependent production rate of CX43	0.02 min^{-1}	Assumed
j_{sCX431}	Michaelis constant of HIF-1α-aOH-dependent production of CX43	6	Assumed
k_{sCx432}	HIF-1α-dependent production rate of CX43	0.02 min^{-1}	Assumed
j_{sCx432}	Michaelis constant of HIF-1α-dependent production of CX43	6	Assumed
d_{Cx43}	Degradation rate of Cx43	0.005 min^{-1}	Assumed
k_1	Pyruvate-dependent transition rate of ADP to ATP	0.1 min^{-1}	Assumed
k_2	Acetyl-CoA -dependent transition rate of ADP to ATP	5 min^{-1}	Assumed
AXP_t	Total concentration of AXP	10	Assumed
d_{ATP}	Degradation rate of ATP	1 min^{-1}	Assumed
d_{ADP}	Degradation rate of ADP	0.4 min^{-1}	Assumed
d_{AMP}	Degradation rate of AMP	0.3 min^{-1}	Assumed
k_{outATP}	Cx43-dependent out-flow rate of ATP	0.3 min^{-1}	Assumed
d_{ATPout}	Degradation rate of extracellular ATP	1 min^{-1}	Assumed
$k_{tsATPout}$	CD73-dependent conversion rate of ATP to Adenosine	0.2 min^{-1}	Assumed
k_{sCD730}	Basal production rate of CD73	0.0002 min^{-1}	Assumed
k_{sCD731}	HIF-1α-aOH-dependent production rate of CD73	0.02 min^{-1}	Assumed
j_{sCD731}	Michaelis constant of HIF-1α-aOH-dependent production of CD73	4	Assumed
k_{sCD732}	HIF-1α-dependent production rate of CD73	0.01 min^{-1}	Assumed
j_{sCD732}	Michaelis constant of HIF-1α-dependent production of CD73	6	Assumed

SUPPLEMENTAL TABLE S2-CONTINUED

Variable	Description	Value	Reference
d_{CD73}	Degradation rate of Cd73	0.005 min^{-1}	Assumed
$d_{Adenosine}$	Degradation rate of Adenosine	0.2 min^{-1}	Assumed
k_{SA2B0}	Basal production rate of A2B	0.0002 min^{-1}	Assumed
k_{SA2B1}	HIF-1α-aOH-dependent production rate of A2B	0.02 min^{-1}	Assumed
j_{SA2B1}	Michaelis constant of HIF-1α-aOH-dependent production of A2B	4	Assumed
k_{SA2B2}	HIF-1α-dependent production rate of A2B	0.02 min^{-1}	Assumed
j_{SA2B2}	Michaelis constant of HIF-1α-dependent production of A2B	6	Assumed
d_{A2B}	Degradation rate of A2B	0.005 min^{-1}	Assumed
k_{acA2B}	Adenosine-dependent activation rate of A2B	1 min^{-1}	Assumed
$j_{Adenosine}$	Threshold of Adenosine for promoting activation of A2B	5	Assumed
j_{acA2B}	Threshold of A2B for its activation	2	Assumed
k_{deA2B^*}	Deactivation rate of A2B*	1 min^{-1}	Assumed
j_{deA2B^*}	Threshold of A2B for its deactivation	3	Assumed

REFERENCES

[1] Qutub, A. A. and A. S. Popel. 2006. A computational model of intracellular oxygen sensing by hypoxia-inducible factor HIF1 alpha. *J Cell Sci* 119(16): 3467-3480.

[2] Koivunen, E., M. Hirsilä, V. Gunzler, K. I. Kivirikko, and J. Myllyharju. 2004. Catalytic properties of the asparaginyl hydroxylase (FIH) in the oxygen sensing pathway are distinct from those of its prolyl-4-hydroxylases. *J Biol Chem* 279: 9899-9904.

[3] Wang, P., Guan, D., Zhang, X.-P., Liu, F. and Wang, W. 2019. Modeling the regulation of p53 activation by HIF-1 upon hypoxia. *FEBS Lett*, 593: 2596-2611.

[4] Daleprane, J. B., T., Schmid, N., Dehne, M., Rudnicki, H., Menrad, T., Geis, & D. S. Abdalla. 2012. Suppression of hypoxia-inducible factor-1α contributes to the antiangiogenic activity of red propolis polyphenols in human endothelial cells. *J Nutr*, 142(3): 441-447.

[5] Bagnall, J., J., Leedale, S. E., Taylor, D. G., Spiller, M. R., White, K. J., Sharkey, & V. Sée. 2014. Tight control of hypoxia-inducible factor-α transient dynamics is essential for cell survival in hypoxia. J Biol Chem, 289(9): 5549-5564.

[6] Dao, D. T., L., Anez-Bustillos, A., Pan, A. A., O'loughlin, P. D., Mitchell, G. L., Fell, & C. J. Smithers. 2018. Vascular Endothelial Growth Factor Enhances Compensatory Lung Growth in Piglets. *Surgery*, 164(6): 1279-1286.

[7] Frazier, D. P., A., Wilson, R. M., Graham, J. W., Thompson, N. H., Bishopric, & K. A. Webster. 2006. Acidosis regulates the stability, hydrophobicity, and activity of the BH3-only protein Bnip3. *Antioxid Redox Sign*, 8(9-10): 1625-1634.
